# Quasi-3D Plasmonic Metamaterials with Highly Stretch-Tunable Optical Responses

**DOI:** 10.1021/acsami.5c22436

**Published:** 2026-01-27

**Authors:** I-Chen Chen, Yu-Chi Huang, Wei-Ting Chao, Yung-Ling Kao, You-Le Lin, Kun-Lung Liao

**Affiliations:** Institute of Materials Science and Engineering, 34911 National Central University, Zhongli 320, Taiwan

**Keywords:** gallium nanoparticles, liquid metal nanocomposites, 3D stacked nanoparticles, active plasmonic metamaterials, plasmon polaritons

## Abstract

Three-dimensional (3D) assemblies of metal nanoparticles (NPs) exhibiting strong plasmonic responses have garnered significant interest owing to their potential in optoelectronic and sensing applications. However, the realization of active plasmonic metamaterials based on such architectures remains nontrivial, particularly in achieving uniform and sub-10 nm interparticle spacing within dynamically tunable media. Here, we present mechanically reconfigurable plasmonic nanocomposites composed of 3D stacked liquid gallium nanoparticles (GaNPs) embedded in a polydimethylsiloxane (PDMS) matrix, fabricated through a single-step Ga evaporation process. The resulting GaNPs/PDMS nanocomposites exhibit multilayered NP architectures with narrow interparticle spacing that can act as quasi-3D plasmonic metamaterials, where collective plasmon resonances hybridize with cavity modes to generate plasmon–polariton states. Under applied biaxial strain, the multilayered architecture enables simultaneous modulation of both intralayer and interlayer plasmonic coupling, giving rise to a reversible strain-induced spectral shift exceeding 300 nm. Finite-difference time-domain (FDTD) simulations confirm that the observed resonance shifts primarily originate from variations in both intra- and interlayer interparticle spacing. Based on structural characterization, furthermore, we propose a refined mechanism for the growth and embedment of nanoparticles within the polymer matrix. These findings could advance the understanding of nanoparticle–polymer interactions and benefit the development of mechanically tunable plasmonic metamaterials.

## Graphical abstract

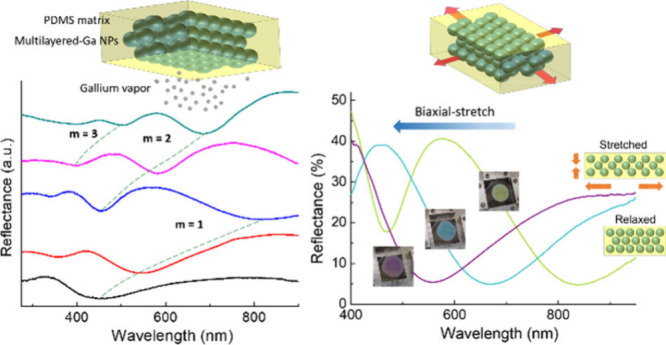

## Introduction

Plasmonic metamaterials are artificially engineered materials composed of periodically or randomly distributed nanostructures with the size and spacing smaller than the wavelength of incident light.^1–3^ Due to their ability to manipulate electromagnetic radiation and enhance light–matter interactions,^4–7^ plasmonic metamaterials have emerged as an attractive research field in nanophotonics. Among various plasmonic nanostructures, the cost-effective fabrication methods and unique optical properties of metal nanoparticles (NPs) make them potentially useful as building blocks for novel metamaterials. Recently, liquid gallium (Ga) has gained increasing attention as an emerging plasmonic material, owing to its nearly free-electron Drude behavior spanning a broad spectral range from the ultraviolet to the infrared.^8^ In addition, GaNPs exhibit distinctive attributes, including high metal solubility and relatively low toxicity, making them highly promising for diverse functional and plasmonic applications.^9–14^

Colloidal metal NPs can be self-assembled into different architectures from one-dimensional (1D) chains to three-dimensional (3D) networks, giving rise to new collective optical responses that originate from plasmonic coupling of metamaterial constituents separated by small gaps.^15–21^ Because the collective plasmonic behavior is highly sensitive to nanoparticle size, shape, interparticle distance and the surrounding medium, earlier studies primarily focused on tailoring the optical response of NP arrays with synthetically tunable, yet permanent structural parameters. More recently, research has shifted toward integrating dynamic components into otherwise passive metallic nanostructures, leading to the development of active plasmonic metamaterials capable of reversible modulation of plasmon resonances in response to external stimuli such as chemical environment, temperature, mechanical strain or humidity.^22–27^ Mechanically reconfigurable metamaterials, achieved by dispersing metal NPs within polymers, have been extensively investigated for plasmonic applications. In these metamaterials, the interparticle spacing between NPs and their assembled structures are crucial factors that determine their plasmonic properties and tunability. Solution-based self-assembly techniques have been widely employed to organize metal nanoparticles (NPs) into 1D and two-dimensional (2D) architectures on polymer substrates. These plasmonic nanostructures exploit the intrinsic finite interparticle spacing to enable dynamically reconfigurable, strain-responsive optical behavior.^28–31^ However, reconfigurable plasmonic composites incorporating 3D stacked NP architectures have been scarcely explored, as achieving uniform interparticle spacing within 3D assemblies embedded in polymers remains a considerable challenge.

Herein, liquid gallium NPs/polydimethylsiloxane (GaNPs/PDMS) nanocomposite films comprised of layer-by-layer self-assembly of liquid GaNPs embedded into the PDMS matrix were obtained through single-step gallium metal evaporation. This simple yet scalable fabrication method enables the formation of multilayered GaNPs with narrow interparticle spacing. A reversibly tunable resonance wavelength shift of more than 300 nm can be achieved by applying biaxial stress on the nanocomposite. Recently, a related study employed a similar fabrication approach to prepare Ga nanostructures embedded in PDMS and demonstrated strain-tunable chromaticity under uniaxial deformation.^32^ While that study proposed a growth model and focused primarily on color tunability, the present work provides several key advances that deepen the understanding of the growth mechanism and light–matter interactions in multilayered GaNPs/PDMS nanocomposites. Specifically, we uncover the emergence of plasmon polariton behavior in the multilayered architectures, revealing previously unexplored light−matter interactions in these flexible nanocomposites. Based on structural characterization, we further propose an alternative growth mechanism, offering a revised interpretation of nanoparticle formation within elastomeric matrices. Moreover, our simulations indicate that, beyond intralayer interactions, interlayer coupling plays a non-negligible role in governing the strain-dependent optical response of the stacked nanoparticle architecture.

## Results and Discussion

### Fabrication and Characterization of Materials

The morphology and surface topography of GaNPs/PDMS nanocomposite thin films were investigated as a function of the Ga deposition rate. For comparison, GaNPs were also deposited on Si substrates. Scanning electron microscope (SEM) images (Figure 1a,e) show that hemispherical GaNPs are randomly distributed on the Si surface, exhibiting a bimodal size distribution arising from surface diffusion and Ostwald ripening, as reported in our previous study.^33^ By contrast, GaNPs deposited on PDMS at a low deposition rate of 0.3 Å/s display a markedly narrower size distribution and a compact arrangement (Figure 1b). The average particle diameter and interparticle spacing (surface-to-surface) were found to be 56.7 ± 0.9 nm and 8.4 ± 0.2 nm, respectively (Figure S1). Furthermore, for GaNPs deposited on PDMS at 0.3 Å/s, the average particle diameter remains essentially unchanged with increasing deposition thickness (Figure 2a), while GaNPs deposited on Si exhibit progressive particle coarsening and a broadened size distribution as the deposition thickness increases (Figure S2). These observations suggest that GaNPs could form within the PDMS matrix under low-rate deposition conditions, enabling self-assembly of GaNPs into multilayer architectures. Transmission electron microscopy (TEM) provides direct structural evidence for this interpretation. As shown in Figure 1f, multilayered GaNP structures are clearly resolved at a deposition rate of 0.3 Å/s (Figure 1f), and the number of stacked NP layers increases with increasing deposition thickness (Figure 2b). Elemental mapping further reveals that silicon, a constituent element of PDMS, is distributed throughout the multilayered NP region (Figure 2c), confirming that the GaNPs are embedded beneath the PDMS surface. Additional support is obtained from NaOH etching experiments: GaNPs deposited on glass substrates are readily dissolved by the etchant, whereas GaNPs deposited on PDMS remain intact after etching, providing further evidence for the embedding of GaNPs within the PDMS matrix.

**Figure 1:**
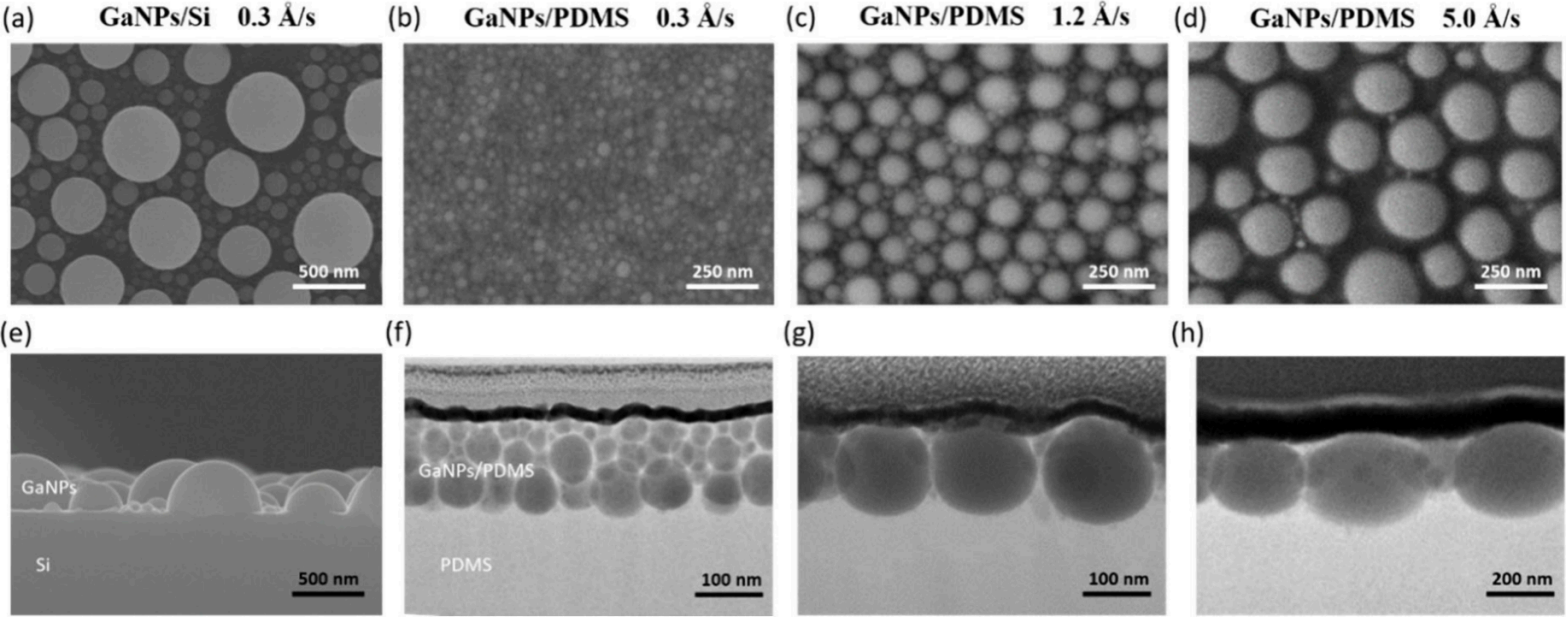
Thermal evaporation of gallium metal with a deposition thickness of 120 nm on PDMS and Si substrates. (a) Top-view and (e) cross-sectional SEM images of GaNPs deposited on Si with a deposition rate of 0.3 Å/s. (b–d) Top-view SEM images and (f–h) cross-sectional TEM images of GaNPs deposited on PDMS with deposition rates of 0.3, 1.2, and 5 Å/s, respectively.

**Figure 2:**
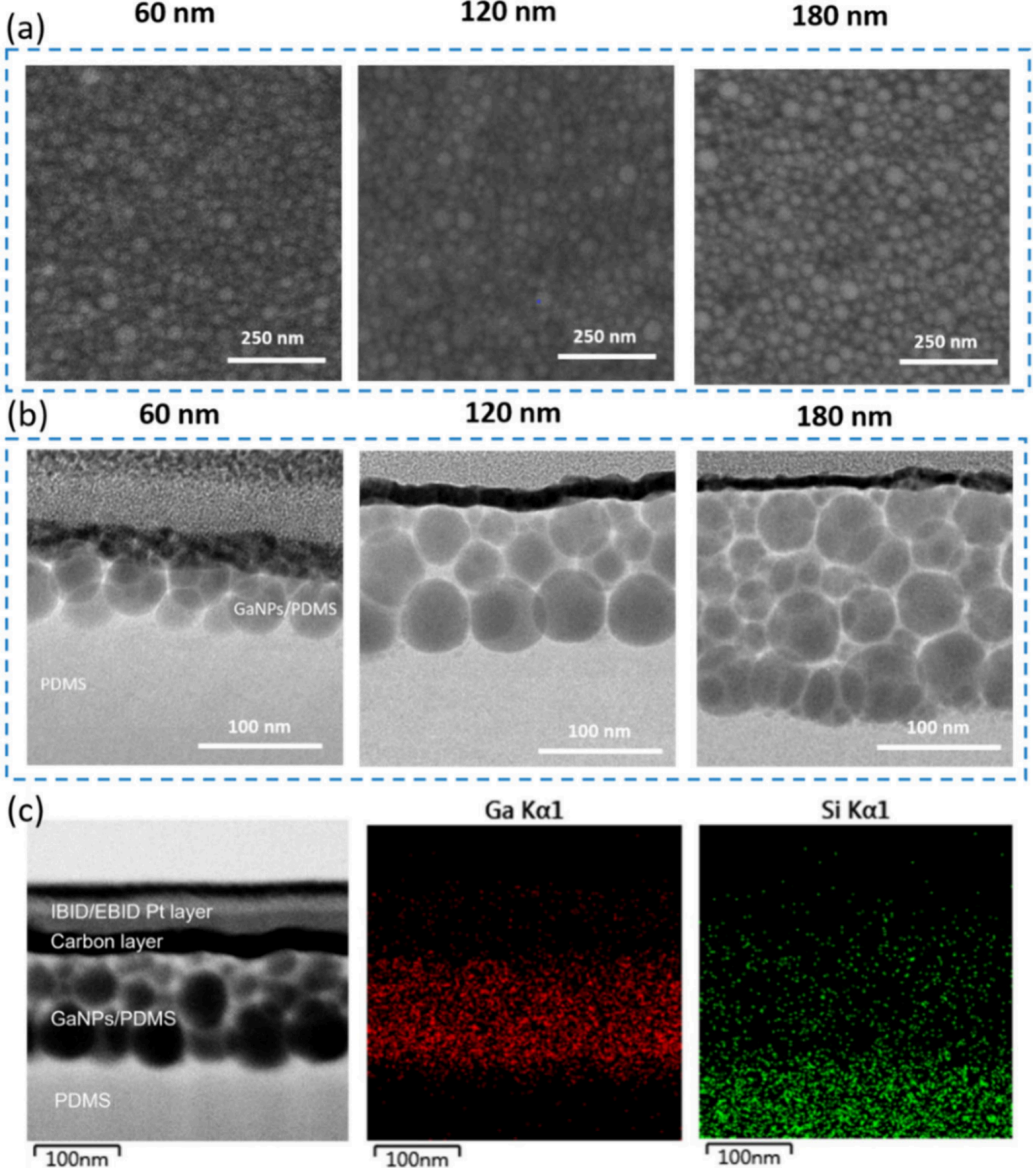
(a) Top-view SEM images and (b) cross-sectional TEM images of GaNPs deposited on PDMS substrates at a deposition rate of 0.3 Å/s with various deposition thicknesses (60, 120, and 180 nm). (c) FIB cross-sectional TEM image of a representative GaNPs/PDMS nanocomposite film and corresponding EDS elemental mapping images.

Nanocomposite layers comprising noble metal NPs, such as Cu, Ag and Au, embedded beneath the surface of thermoplastic polymers have been fabricated by metal evaporation at substrate temperatures exceeding the glass transition temperature (*T*_g_) and at extremely low deposition rates (<0.5 monolayers/min).^34–36^ The segmental motions within the polymer matrix predominantly take place above *T*_g_, facilitating the subsurface migration of metal NPs through localized rearrangement of polymer chains.^34,37^ The incorporation of Au and Ga NPs into PDMS have also been demonstrated via vacuum evaporation.^32,38^ The previous studies suggest that un-cross-linked oligomers diffuse outward from the bulk PDMS toward the NP surface, subsequently encapsulating the growing nanoparticles. A theoretical model based on the interplay between oligomer migration and NP growth kinetics was further proposed to describe the formation of multilayered GaNPs.^32^ While we acknowledge that oligomer mobility and concentration play critical roles in governing the morphological evolution of GaNPs, our results suggest that the formation of the NP-embedded structure arises primarily from the high self-diffusion of Ga atoms within liquid GaNPs, which promotes nanoparticle growth inside the PDMS matrix, coupled with oligomer-assisted encapsulation—rather than solely from outward oligomer diffusion. To evaluate this hypothesis, Ga was deposited onto PDMS substrates pretreated by hexane extraction for 24 h to remove excess oligomers. The TEM image (Figure S3) reveals that the embedded NP structure persist in the absence of oligomers. Due to its exceptionally low *T*_g_ (less than – 100 °C) and high free volume fraction,^39^ PDMS is expected to exhibit long-range segmental mobility. Such intrinsic mobility may facilitate nanoparticle penetration and encapsulation even without the assistance of oligomers.

Furthermore, Au was deposited on PDMS for comparison since both Au and Ga possess relatively high surface energies (Au, ∼1500 mJ m^–2^; Ga, ∼720 mJ m^–2^).^40,41^ Under the deposition conditions of a deposition rate of 0.3 Å/s and a deposition thickness of 120 nm, the Au/PDMS sample (φ=8:1) displays the golden color of bulk gold (Figure 3a), indicating the formation of a continuous thin film on the PDMS surface, as confirmed by four-point probe measurements (sheet resistance: ∼ 90 ohm/sq). Because the deposition conditions for Au and Ga are identical, if the oligomer mobility were the sole determining factor, deposited Au would also be expected to form an embedded structure. Based on the above experimental observations, we infer that the high self-diffusion coefficient of liquid Ga is a key factor responsible for the morphological differences between Ga and Au deposition. A PDMS substrate with φ = 8:1 contains a higher proportion of curing agent than the standard 10:1 formulation, resulting in an excess of cross-linker that behaves as a liquid-like oligomer. To the best of our knowledge, no studies have reported the diffusion coefficient of the cross-linker species in cured PDMS. However, the diffusion coefficients of liquid *n*-alkanes (n = 5–10) in cured PDMS at room temperature are on the order of 10^–7^ cm^2^ s^–1^.^42^ Given that the molecular weight of the cross-linker is considerably higher than that of *n*-alkanes, we estimate that the diffusion coefficient of the excess cross-linker (D_cr_) in PDMS (φ = 8:1) is smaller than 10^–7^ cm^2^ s^–1^. Consequently, the deposition rate (V_dep_) is expected to greatly exceed the cross-linker migration rate (V_migr_) under these conditions. In the initial stage of Ga deposition, small Ga clusters form and become partially embedded within the PDMS surface because of its large elastocapillary length. Due to the liquid nature of Ga and its high self-diffusion coefficient (D_Ga_ ∼ 1.4 × 10^–5^ cm^2^/s at room temperature),^43,44^ we propose that, during NP growth, the deposited Ga atoms readily diffuse into the interior of the liquid GaNPs. This inward diffusion promotes nanoparticle expansion predominantly toward the PDMS matrix rather than the air interface (Figure 3b), as the inward growth effectively minimizes the surface energy of the system. Simultaneously, a cross-linker wetting layer progressively engulfs the partially exposed GaNPs through the diffusion flux of cross-linker chains. In contrast, atomic diffusion in bulk Au is negligible at room temperature (D_Au_ ≪ 10^–12^ cm^2^/s);^45^ thus, small Au clusters on PDMS tend to grow isotropically above the surface (Figure 3b). As a result, the Au islands coalesce into a continuous film, driven by predominant surface accumulation and surface diffusion of Au adatoms that outpace the migration of cross-linker chains. Au and Ga depositions were also carried out on PDMS substrates prepared with φ = 3:1, which contain a large fraction of unreacted cross-linkers, leading to a high cross-linker migration rate (V_migr_ > V_dep_).^38^ Under identical deposition conditions, the Au/PDMS sample (φ = 3:1) exhibits a distinct purple coloration (Figure 3a), characteristic of surface plasmon resonance, indicating the formation of embedded Au nanostructures within the PDMS matrix. In the GaNPs/PDMS system, the NP-embedded structure is still observable even at deposition rates as high as 5 Å/s. At the higher deposition rates, a monolayered GaNP structure is obtained (Figure 1g, h) in contrast to the multilayered architectures formed at a lower rate of 0.3 Å/s (Figure 1b). The average size of GaNPs increases with increasing the deposition rate (Figure 1c, d), with particle diameters exceeding 150 nm at 5 Å/s. Further investigation is required to elucidate the underlying mechanism governing the development of densely packed, multilayered GaNPs beneath the PDMS surface.

**Figure 3:**
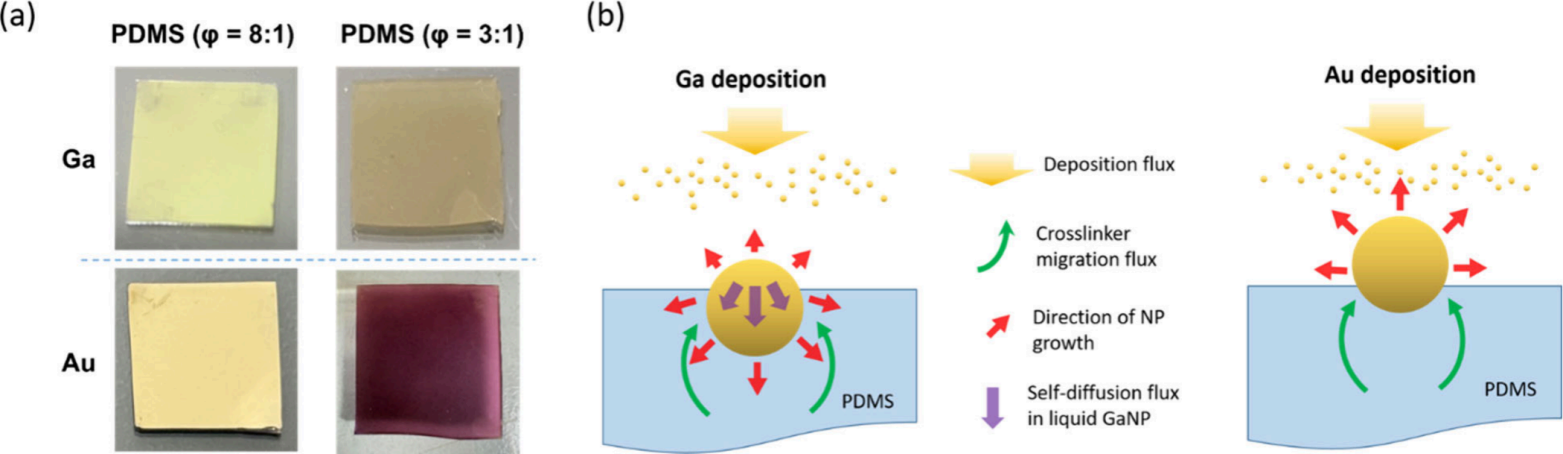
(a) Photo images of samples prepared by thermal evaporation of Ga and Au metals on PDMS substrates. (b) Schematic illustration of the formation of Ga and Au nanoparticles during thermal evaporation.

### Optical Properties of GaNPs/PDMS Nanocomposite Thin Films

The reflectance spectra of GaNPs/PDMS nanocomposites were measured as a function of the deposition thickness. We compared the optical responses between monolayered and multilayered GaNPs/PDMS nanocomposite films prepared at deposition rates of 1.2 Å/s and 0.3 Å/s, respectively (Representative results are shown in Figures 4a and 4b). By using a higher deposition rate of 1.2 Å/s, a monolayered NP-embedded structure is maintained with an increased particle size and broader size distribution as the deposition thickness increases (Figure S4). Only one prominent reflectance dip is clearly observed in the monolayered-GaNPs/PDMS system (Figure 4a), which is indicative of the transverse (intralayer) coupling between NPs. The increased NP diameter leads to a red-shifted and widened plasmonic reflectance dip due to the interface damping and phase retardation effects.^46,47^ The broader size distribution accompanied by larger NPs also causes additional line width broadening.^48^ In contrast, in the multilayered GaNPs/PDMS system, a reflectance dip (denoted by order number m = 1) begins to appear around 450 nm with a deposition thickness of 60 nm, corresponding to the initial formation of a bilayered NP structure beneath the PDMS surface (Figure 2b). As the deposition thickness increases, more reflectance dips appear, systematically redshifting to longer wavelengths (Figure 4b). Furthermore, the process reproducibility was evaluated through spectral measurements. The nearly identical optical spectra obtained from multiple deposition runs (Figure S5) imply that the NP size and interparticle spacing distributions are highly consistent across different deposition runs.

**Figure 4:**
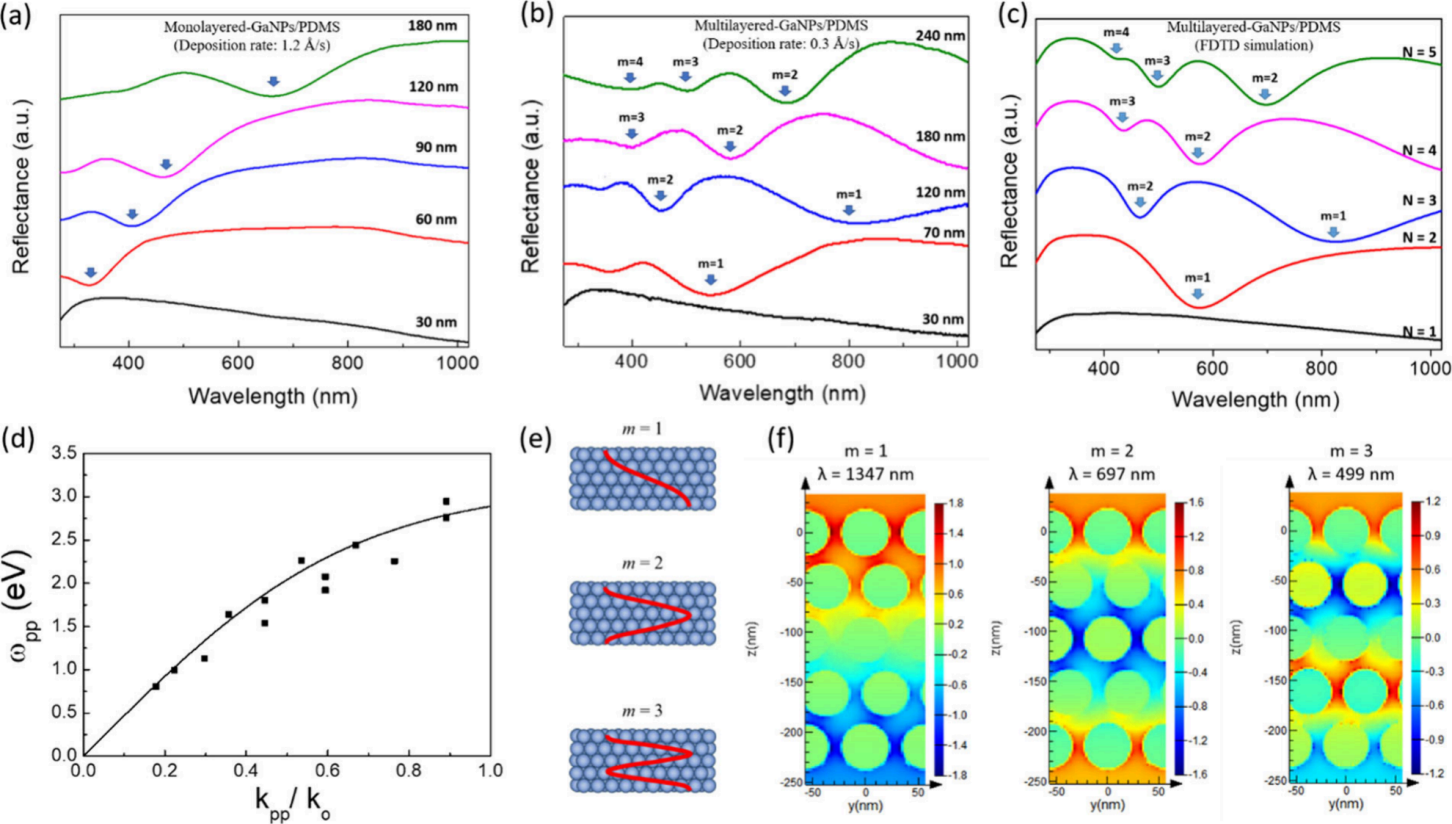
(a and b) Measured reflectance spectra of monolayered and multilayered GaNPs/PDMS nanocomposite films, respectively, as a function of the deposition thickness. (c) Simulated reflectance spectra of perfectly ordered multilayered GaNPs/PDMS nanocomposites as a function of the layer number (N). Arrows indicate the major dips associated with polariton resonances. (d) Measured plasmon polariton dispersion of the multilayered GaNPs/PDMS nanocomposites. The solid line is the fit of the Hopfield model (eq 2). (e) Schematic of standing waves inside a 3D NP crystal. (f) Simulated electric field distribution inside a five-layer GaNPs/PDMS nanocomposite.

To gain deeper insight into the origin of multiple plasmonic dips, the reflectance spectra of multilayered-GaNPs/PDMS composites were simulated using the FDTD approach. Previous studies have shown that imperfections in 2D nanoparticle lattices, such as minor variations in the NP size and positional displacement, only exert minimal influence on the optical properties.^49,50^ Therefore, a hexagonal lattice model with the average NP diameter and interparticle spacing (obtained from histogram analysis in Figure S1) was adopted for FDTD simulation. Due to the high oxygen permeability of PDMS,^51,52^ vacuum-deposited GaNPs are readily oxidized on exposure to air, leading to the formation of a self-limiting native oxide shell, as confirmed by the XPS spectra (Figure S6a). Literature reports indicate that the shell thickness typically ranges from 1 to 3 nm.^53–55^ The influence of this surface oxidation on the optical resonance of the nanocomposite was further evaluated by FDTD simulation (Figure S6b). In the following simulations, the shell thickness was assumed to be 1 nm. Figure 4c shows the calculated reflectance spectra for 3D hexagonally packed lattices of GaNPs embedded 10 nm beneath the PDMS surface with the number of NP layers (N) ranging from 1 to 5. Comparison between the experimental and simulated spectra (Figure 4b and 4c) reveals that both the dip positions and their shifting trends are in quantitative agreement, enabling the assignment of the major spectral dips to resonance modes with m = 1–4. Few minor dips observed in the ultraviolet region of the measured spectra (Figure 4a) are absent in the simulations. This discrepancy may arise from structural imperfections in the topmost GaNP layer, where short-wavelength light interacts most strongly.

A similar optical resonance feature has also been experimentally observed in plasmonic supercrysals made by 3D assembly of Au NPs on substrates via solution-based transfer processes.^56–58^ Such plasmonic crystals exhibit Fabry–Perot cavity modes which can couple with collective plasmonic states, leading to the optical excitation of plasmon polaritons. The propagation of plasmon polaritons is confined inside the crystal, which results in the formation of standing waves (Figure 4e) and thus a series of spectral reflectance dips. In this study, although the GaNPs do not assemble into a fully ordered structure, we suggest that the GaNPs/PDMS nanocomposite can also act as a cavity that supports plasmon polariton modes. To examine the light-matter coupling in the GaNPs/PDMS nanocomposite, the dispersion of reflectance dips was extracted from the experimental spectral results and analyzed by the theoretical simulation. In this study, given that GaNPs are not perfectly stacked in a layer-by-layer fashion within the PDMS matrix, the polariton wavevector (*k*_*pp*_) corresponding to the *m*th resonance mode in the measured spectra can be expressed as

1 $$k_{pp}=m\pi /t_{dep}$$

where the deposition thickness *t*_*dep*_ is approximately equal to the cavity length. For the simulation of the dispersion relation, we consider a FCC lattice of GaNPs built by stacking along the (111) direction. The extracted dispersion is plotted versus *k*_*pp*_ normalized by k_ΓL_, the length of the first Brillouin zone in the ΓL direction (Figure 4d). We adopt the calculation approach in ref 57. and fit the extracted dispersion with the Hopfield Hamiltonian, yielding a dispersion relation for hybridized plasmon polariton modes ω_pp_(k),

2 $${\omega_{pp}}^{4}\left(k\right)-{\omega_{pp}}^{2}\left(k\right){\omega_{pt}}^{2}\left(k\right)+{\omega_{pl}}^{2}\left(k\right)+4{\Omega_{R}}^{2}+{\omega_{pt}}^{2}\left(k\right){\omega_{pl}}^{2}\left(k\right)=0$$

with the photon energy ω_pt_, the transverse plasmon frequency ω_pl_ and the Rabi frequency Ω_R_. The fitting parameters are listed in Table S1. Here, the oxide shell of GaNPs is ignored in the Hopfield Hamiltonian model. The measured dispersion relation is in reasonable agreement with the calculated polariton dispersion of the perfectly ordered GaNPs/PDMS system (Figure 4d). The upper polariton branch is not observable experimentally because it is beyond the spectral range (λ < 270 nm) of our spectrometer (Figure S7). Furthermore, we also examine the spatial distribution of the electric field in orderly stacked GaNPs/PDMS nanocomposites using FDTD simulations. Figure 4f presents the electric-field amplitude in a five-layer GaNPs/PDMS system at wavelengths corresponding to reflectance dips. The simulations clearly reveal the standing-wave nature of the electric field.

### Dynamic Plasmon Tuning of GaNPs/PDMS Nanocomposite Thin Films

A pronounced red-shift of the m = 1 resonance mode is observed with increasing deposition thickness (Figure 4b), indicating its high sensitivity to structural variations in the multilayered GaNPs/PDMS nanocomposites. Thus, the *m* = 1 mode was selected as a probe to evaluate the plasmonic tunability of the system under mechanical stimuli. Our central objective was to realize an optical metamaterial architecture with high mechanical reconfigurability across the visible spectrum. For this purpose, the GaNPs/PDMS nanocomposite film with t_dep_ = 120 nm was chosen since its m = 1 mode can be tuned over the visible and near-infrared regions under in-plane stress. Mechanical deformation was applied using a home-built biaxial stretching apparatus (Figure S11), and the evolution of the *m* = 1 reflectance dip was recorded at different stretch ratios (ε). To quantitatively evaluate the deformation induced by stretching, the projected area of the GaNPs pattern was extracted from the optical images before and after stretching using ImageJ software, and the relative change in projected area was employed as a measure of the overall stretching-induced deformation. Although local strain variations are expected within the patterned region under mechanical loading, the projected area change provides an effective estimate of the average in-plane strain over the optically active area, which is directly responsible for the observed plasmonic response. Here, the stretch ratio (ε) is defined as the ratio of the projected area change to the original projected area. It is also noted that the thickness of the GaNPs/PDMS patterned layer (∼120 nm) is negligible compared to that of the bulk PDMS substrate (∼0.8 mm), and therefore the patterned layer is not expected to significantly affect the overall mechanical response of the sample. A significant blue-shift of the m = 1 reflectance dip (from 838 to 517 nm) was experimentally obtained as the stretch ratio increased from 0% to 88% (Figure 5a), which demonstrates a relatively extensive tunability (>300 nm) compared with the previous reported values for stretch-tunable plasmonic structures, such as 160 nm for Ag cubes/PDMS and 150 nm for Al NPs/PDMS.^27,59^ Accordingly, an obvious change of color was observed by adjusting the stretch ratio (Figure 5b). The corresponding visible color varied from light yellow to blue and eventually to a reddish-purple. This color shift is further represented as color coordinates in the CIE1931 plot (Figure 5c). Despite the relatively small occupied area on the CIE plot, the generated colors are distinct and positioned around the achromatic point in the CIE color space, which is crucial for developing a system capable of displaying all primary colors. We suggest that the remarkable blue-shift of the reflectance dip in the biaxially stretched nanocomposite is primarily governed by the extent of interparticle coupling and the polymer matrix. In parallel to the experimental measurements, theoretical calculations were conducted to elucidate the effects of interparticle spacings and the polymer matrix on the optical properties of the nanocomposite film.

**Figure 5:**
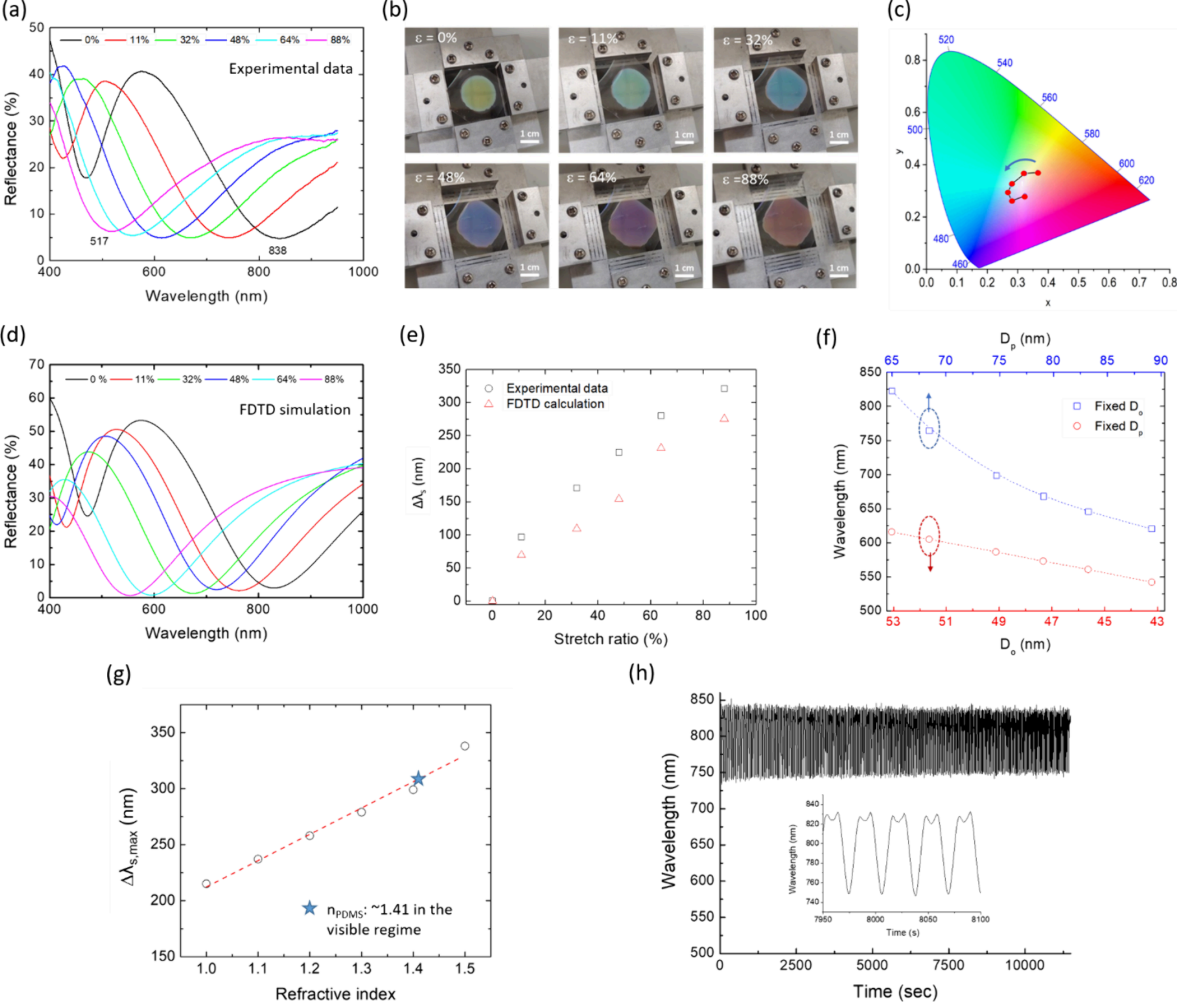
(a) Measured reflectance spectra of a representative multilayered GaNPs/PDMS nanocomposite film (*t*_dep_ = 120 nm) under various biaxial stretch ratios. (b) Optical images and (c) CIE color coordinates of the representative GaNPs/PDMS nanocomposite film under various biaxial stretch ratios. (d) Simulated reflectance spectra of three-layer GaNPs embedded beneath the PDMS surface at various stretch ratios. (e) Comparison of experimentally measured and simulated Δλ_s_ values as a function of stretch ratio. (f) Simulated *m* = 1 resonance wavelength as a function of the *D*_p_/*D*_o_ ratio under conditions of fixed *D*_p_ and fixed *D*_o_, respectively, as shown in Figure S9. (g) Simulated Δλ_s,max_ as a function of the surrounding medium refractive index. The dashed line denotes a linear fit. (h) Reversible plasmonic tunability of the representative GaNPs/PDMS nanocomposite film over 500 stretching–releasing cycles.

The three-layer GaNPs/PDMS structure was employed for FDTD simulations of the nanocomposite film under biaxial stretching, owing to its comparable thickness to the as-prepared sample (t_dep_ = 120 nm). We first evaluated whether the GaNPs undergo deformation upon mechanical loading. The deformation of isolated core–shell GaNPs embedded in PDMS under stress depends strongly on the core diameter and the thickness of the outer oxide shell. The Young’s moduli of the PDMS matrix (*E*_*s*_) and the thin Ga_2_O_3_ shell layer (*E*_*ox*_) are reported to be approximately 2 MPa and 30 GPa, respectively.^60–62^ The effective stiffness (*E*_*eff*_) of a spherical core–shell liquid metal NP can be expressed as *E*_*ox*_ *·T*/*R* (where *R* is the core radius and *T* is the oxide shell thickness) under the condition *E*_*ox*_ *·T*/*E*_*s*_ *·R* ≫1.^63^ Using R = 27.5 nm and *T* = 1 nm, we obtain *E*_*ox*_ *·T*/*E*_*s*_ *·R* ≈ 545, yielding an effective stiffness of *E*_*eff*_ ≈ 1.1 GPa for the GaNPs studied here. Given that *E*_*eff*_ /*E*_*s*_ > 500, the core–shell GaNPs can be reasonably regarded as rigid inclusions within the PDMS matrix.^63,64^ Furthermore, we also evaluated the deformation of the liquid Ga nanoparticles (without a native oxide shell) embedded in PDMS. Based on the theoretical model in ref 65., the aspect ratio of liquid Ga droplets remains below 1.05 under an applied uniaxial strain of 0.5 (Figure S8). Therefore, the deformation of GaNPs is considered negligible in the subsequent FDTD simulations, and the stretch-induced displacement of the nanoparticles is assumed to preserve the overall symmetry of the assembly. Upon biaxial stretching of the multilayered-GaNPs/PDMS nanocomposite, the average interparticle distance increases along the stretching direction and decreases in the perpendicular direction. Here, the intralayer spacing (D_p_) and interlayer spacing (D_o_) are defined as the center-to-center distances between adjacent particles parallel and orthogonal to the incident light, respectively (Figure S12). In this study, the in-plane linear displacement is determined from the square root of the projected area ratio, and thus the value of D_p_ can be obtained by

3 $$D_{p}=\sqrt{\left(1+\varepsilon \right)}D_{p,ori}$$

with the original intralayer spacing D_p,ori_ = 65 nm. D_o_ was calculated by assuming a Poisson’s ratio of 0.5 for the GaNPs/PDMS nanocomposite. The corresponding D_p_ and D_o_ values for each stretch ratio are summarized in Table S2. FDTD simulations based on these calculated parameters reveal a pronounced blue-shift of the reflectance dip with increasing the stretch ratio (Figure 5d). The simulated resonance wavelength shift (Δλ_s_), defined as the difference between the relaxed and stretched states, shows good qualitative agreement with the experimental data across various stretch ratios (Figure 5e).

Further modeling was performed to elucidate the respective roles of intralayer (longitudinal) and interlayer (transverse) coupling in the strain-dependent resonance tuning. FDTD simulations of the reflectance spectra were carried out for the three-layer GaNPs/PDMS structure by independently varying D_p_ and D_o_, thereby isolating their individual contributions (Figure S9). The calculated resonance wavelengths under fixed D_p_ and D_o_ conditions were then plotted as functions of D_o_ and D_p_, respectively (Figure 5f). In the case of intralayer coupling with fixed D_o_, a pronounced blue shift of the resonance wavelength (>200 nm) is obtained with increasing D_p_. In contrast, when D_p_ is held constant, modulation of the interlayer coupling through a reduction in D_o_ induces a blue shift of approximately 75 nm. The simulated trends are consistent with both the experimental observations and the theoretical hybridization model for metal NP dimer systems.^66–68^ For comparison, we also performed stretching experiments on a monolayered GaNPs/PDMS nanocomposite, which exhibited a resonance wavelength shift of less than 150 nm at a stretch ratio of 88% (Figure S10). Taken together, the experimental and simulation results suggest that the multilayered GaNPs/PDMS nanocomposites allow simultaneous modulation of both intralayer and interlayer coupling, each contributing to the blue shift of the resonance dip and thereby achieving a broader spectral tunability than that attainable in monolayer NP structures. The effect of the medium refractive index on the plasmon shift was also investigated via FDTD simulation on the three-layer GaNPs structure surrounded by a medium with a refractive index (n) ranging from 1 to 1.5. The calculated Δλ_s,max_ (the plasmon shift between the fully relaxed and 88% stretched cases) increases linearly with an increase in the n value (Figure 5g). The calculation results indicate that compared to an air environment (n = 1), the tunability is effectively enhanced with Δλ_s,max_ increasing from 215 to 300 nm in the presence of PDMS (n ≙1.41 in the visible regime), demonstrating that the surrounding matrix plays a substantial role in strain-induced plasmon tuning of the nanocomposite. We also examined the reversibility of the optical signal in the GaNPs/PDMS nanocomposite by applying a unidirectional cyclic loading at 30% strain. The plasmon shift was found to be fully reversible after 500 repeated stretching/relaxing cycles (Figure 5h), indicating that the structural integrity of the nanocomposite is well preserved.

## Conclusion

In summary, we have demonstrated both experimentally and theoretically a mechanically reconfigurable plasmonic nanocomposite film exhibiting exceptional spectral tunability. The nanocomposite, composed of multilayered assemblies of liquid GaNPs embedded in a PDMS matrix, was fabricated via a single-step gallium evaporation process. By adjusting the deposition rate, either multilayered or monolayered NP architectures can be obtained, enabling control over the dimensionality of plasmonic coupling. The multilayered GaNPs/PDMS nanocomposites behave as quasi-3D plasmonic metamaterials, where collective plasmon resonances hybridize with cavity modes to generate plasmon–polariton states. The tunable plasmonic coupling along both transverse and longitudinal directions under mechanical strain endows these flexible quasi-3D metamaterials with highly dynamic and reversible control over their plasmon resonances. Moreover, the surrounding polymer matrix is proposed to further enhance the mechanical modulation of the plasmonic response in nanoparticle-embedded composite structures. The multilayered GaNPs/PDMS nanocomposites reported here represent a significant advancement in flexible plasmo-mechanical platforms, offering strong potential for next-generation plasmonic and optoelectronic applications.

## Methods

### Sample Preparation

Commercially available PDMS (Sylgard 184, Dow Corning) was used to prepare PDMS substrates. The elastomeric base and curing agent were mixed, thoroughly stirred, and degassed to remove trapped air bubbles. The mixture was then poured into a glass Petri dish and cured in a vacuum oven at 100 °C for 1 h. After curing, the PDMS sheet was peeled off and cut into 30 × 30 mm^2^ pieces. Gallium was subsequently deposited onto the PDMS substrates at room temperature using a thermal evaporator operated at deposition rates between 0.3 and 5 Å/s. The base pressure of the deposition chamber was 3 × 10^–6^ Torr. The deposition thickness was varied in the range of 30–240 nm and controlled by a quartz crystal thickness monitor. Unless otherwise specified, the elastomeric base and curing agent were mixed at a weight ratio (φ) of 8:1.

### Characterization of GaNPs/PDMS Nanocomposite Thin Films

The surface morphology of GaNPs/PDMS nanocomposite films was examined using a field emission scanning electron microscope (FESEM; Hitachi 8220). The cross-sectional transmission electron microscopy (TEM) images and energy dispersive spectroscopy (EDS) elemental mapping images were obtained from a high-resolution transmission electron microscope (JEOL; JEM2100). Reflectance spectra of GaNPs/PDMS nanocomposites at rest and under stress were obtained using the experimental setup shown in Figure S11. All experimental spectra were measured using nonpolarized light and collected with a UV/vis/NIR spectrophotometer. To evaluate the strain-dependent spectral response, biaxial stretching of the nanocomposites was performed using a home-built manual stretcher (Figure S11). Reflectance spectra were recorded during the stretching process with stretch ratios ranging from 0% to 88% of area change. Stretch–release cycling tests were conducted under a uniaxial strain of 30% using a motorized linear stage (X-VSR20A-E01, Zaber Technologies, Inc.) operated at a speed of 8 mm/s.

### Model and Simulations

The FDTD simulation (Ansys–Lumerical software) was performed to model the spectral responses of GaNPs/PDMS nanocomposite films. The simulation structures are mono/multi-layer arrays of nanoparticles embedded in the PDMS matrix with perfectly matched layer (PML) boundary conditions in the incident direction and periodic boundary conditions to its lateral sides. The dielectric constants of liquid Ga and PDMS were taken from refs 69, 70 ., respectively. The hexagonal unit cell model was constructed for multilayered-GaNPs/PDMS nanocomposites as shown in Figure S12. Under a normal incident broadband source, the simulated reflectance spectra were calculated with a frequency-domain monitor located behind the light source.

## Supplementary Material



## Notes

Supplementary optical, SEM, and TEM images; histograms of the particle size distribution and interparticle spacing; measured reflectance spectra of three-layer GaNPs/PDMS nanocomposites obtained from five independent deposition runs; XPS depth profile in Ga 3d core level spectra for the GaNPs/PDMS nanocomposite; simulated reflectance spectra as a function of the oxide shell thickness; simulated plasmon polariton dispersion for orderly stacked GaNPs embedded in PDMS; aspect ratio of liquid Ga droplets in a uniaxially stretched PDMS as a function of strain; simulated reflectance spectra with varied interlayer and intralayer spacing; measured reflectance spectra of the monolayer GaNPs/PDMS nanocomposite film under various biaxial stretch ratios; schematic of the reflectance spectrum measurements; and schematic of the unit cell used for the FDTD simulations (PDF)
