# Supplementary material for: Quasi-3D Plasmonic Metamaterials with Highly Stretch-Tunable Optical Responses
Source: ACS Appl Mater Interfaces. 2026 Jan 27;18(5):8636–45. doi: 10.1021/acsami.5c22436 (PMC12903114; doi:10.1021/acsami.5c22436)
Supplement: Supplementary file 1 [file am5c22436_si_001.pdf]

Supporting Information

Quasi-3D Plasmonic Metamaterials with Highly Stretch-Tunable Optical Responses

I-Chen Chen*, Yu-Chi Huang, Wei-Ting Chao, Yung-Ling Kao, You-Le Lin and Kun-Lung Liao

Institute of Materials Science and Engineering, National Central University, Zhongli 320, Taiwan

***Email:** ichen@ncu.edu.tw

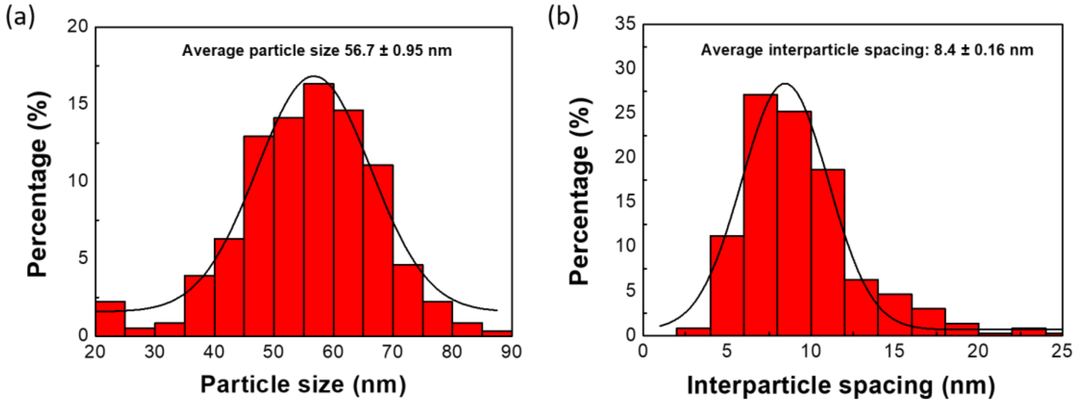

Figure S1. Histogram of (a) particle size distribution and (b) interparticle spacing (surface-to-surface) of GaNPs deposited on PDMS at a deposition rate of 0.3 Å /s.

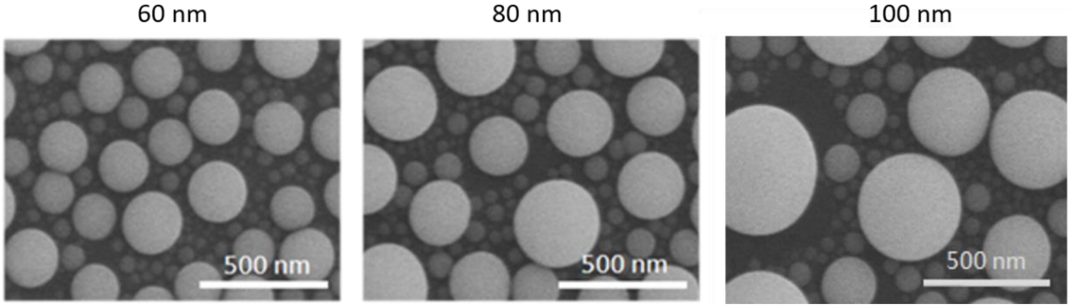

Figure S2. SEM images of GaNPs deposited on Si wafers with deposition thicknesses of 60 nm, 80 nm and 100 nm respectively.

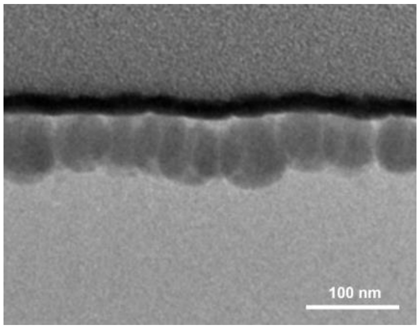

Figure S3. Cross-sectional TEM image of GaNPs deposited on pre-treated PDMS with a deposition thickness of 60 nm. The PDMS substrate was pre-treated by hexane extraction for 24 hours.

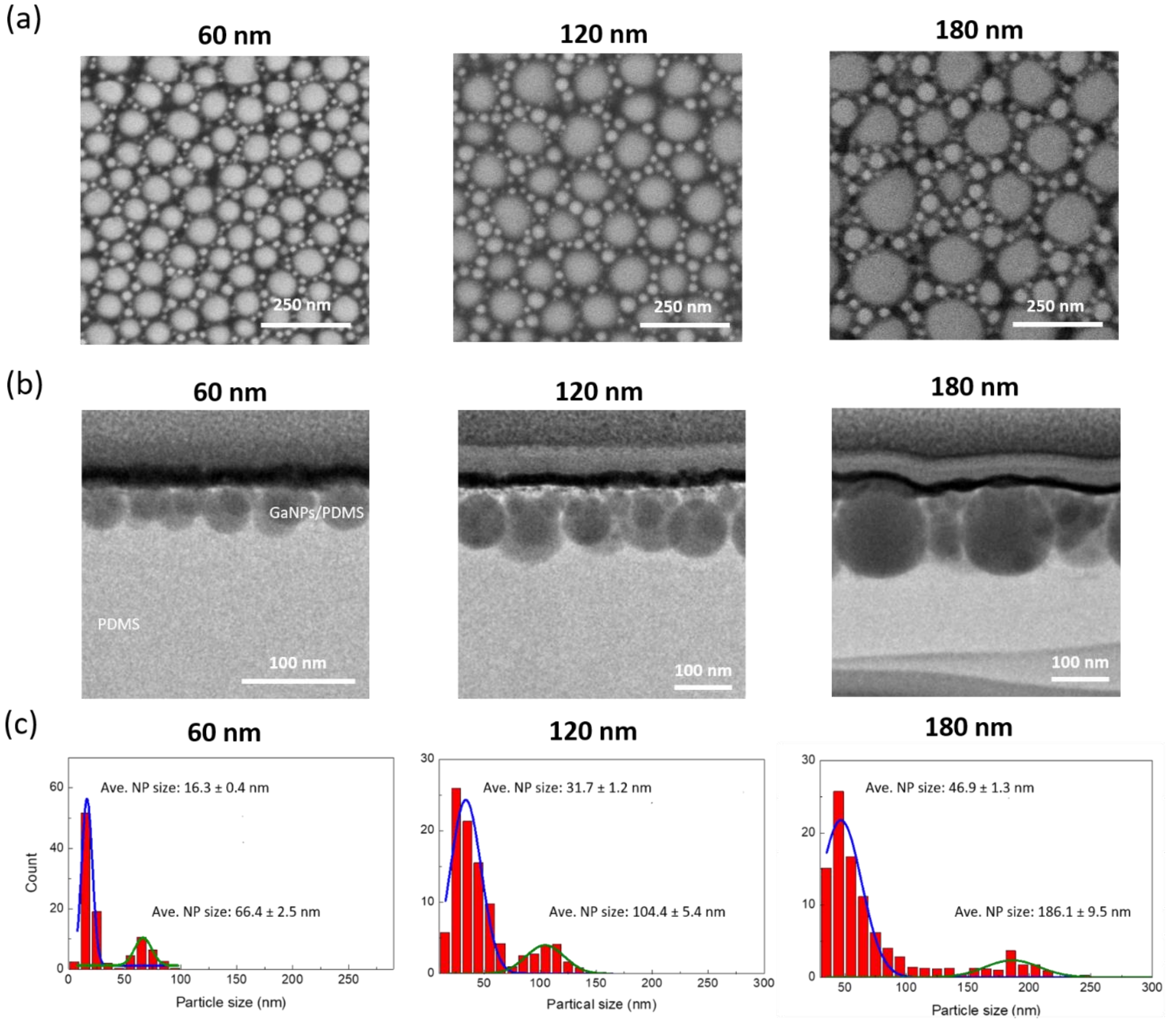

Figure S4. (a) Top-view SEM images, (b) Cross-sectional TEM images and (c) Size distribution histograms of GaNPs deposited on PDMS substrates at a deposition rate of 1.2 Å /s with various deposited thicknesses, respectively.

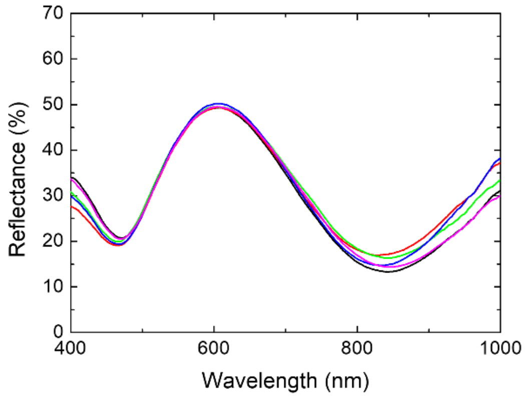

Figure S5. Measured reflectance spectra of three-layered GaNPs/PDMS nanocomposites obtained from five independent deposition runs.

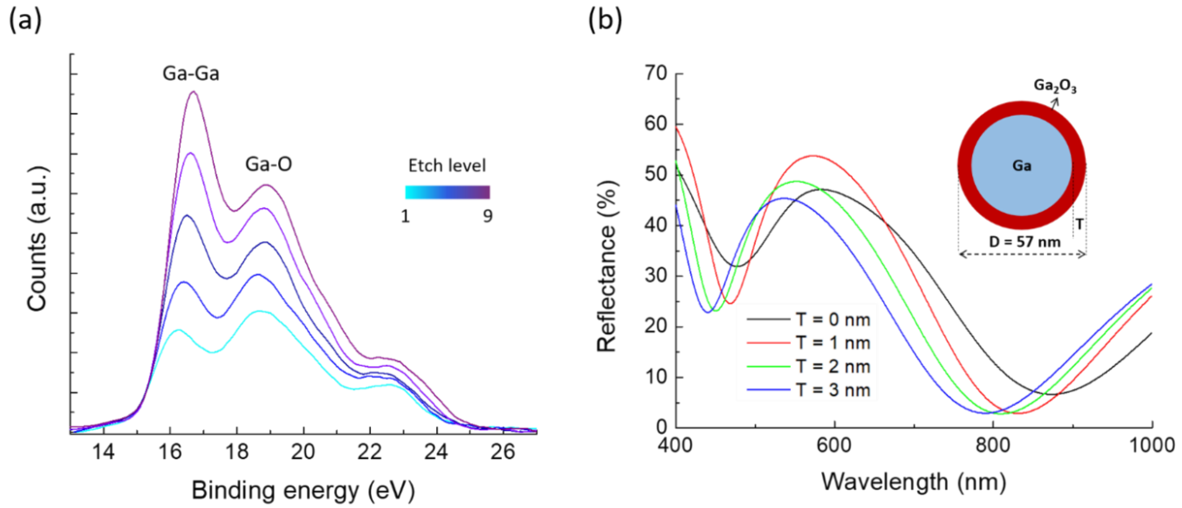

Figure S6. (a) XPS depth profile of Ga-O and Ga-Ga component in Ga 3d core level spectra for a representative GaNPs/PDMS nanocomposite. (b) FDTD simulations of reflectance spectra of the 3-layered GaNPs/PDMS nanocomposite as a function of the oxide shell thickness. The inset shows a side view of the core-shell nanoparticle. For the simulations, the GaNPs are assumed to be with a core-shell spherical structure with a fixed overall diameter but varying the shell thickness. An increment in the shell thickness results in a blue-shift of the resonance dip positions in the calculated reflectance spectra.

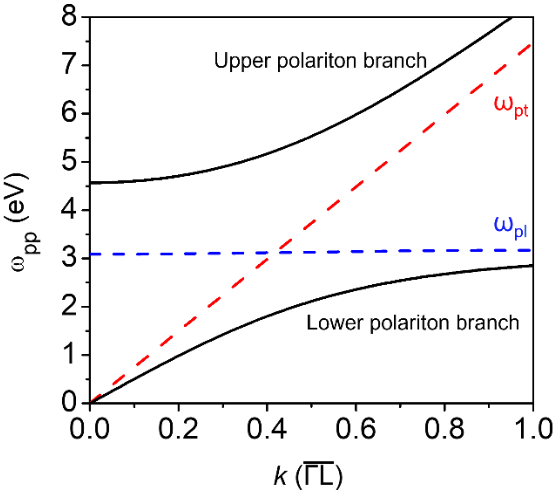

Figure S7. Simulated plasmon polariton dispersion (black lines) along the ΓL direction for orderly stacked GaNPs embedded in PDMS.

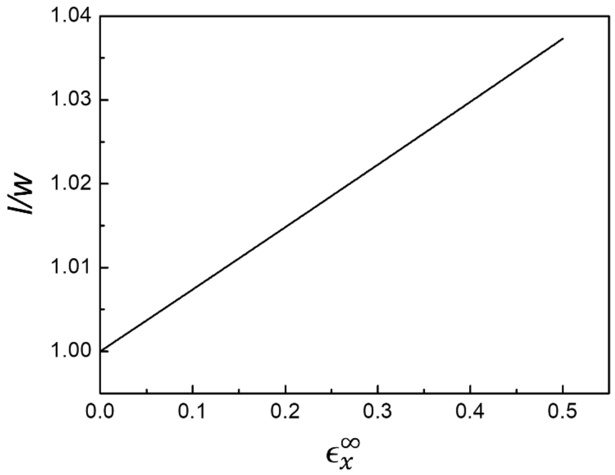

Figure S8. Aspect ratio of liquid Ga droplets (*D* = 55 nm without an oxide shell) in a uniaxially stretched PDMS (E_s = 2 MPa) as a function of strain.

For calculation of the aspect ratio (*l*/*w*) of the droplet, the length (*l*) and width (*w*) of the stretched droplet were determined by[1]

$$l = 2R\left[1 + \frac{5(2\epsilon_1 - \epsilon_2)}{6 + 15\frac{\gamma}{E_s R}}\right] \tag{S1}$$

and

$$w = 2R\left[1 + \frac{5(2\epsilon_2 - \epsilon_1)}{6 + 15\frac{\gamma}{E_s R}}\right] \tag{S2}$$

with $\epsilon_1 = (\epsilon_x^\infty + \nu\epsilon_y^\infty) / (1 - \nu^2)$ and $\epsilon_2 = (\nu\epsilon_x^\infty + \epsilon_y^\infty) / (1 - \nu^2)$, where ν is the Poisson's ratio, and ϵ_x^∞ and ϵ_y^∞ are the applied strain parallel and perpendicular to the stretch direction, respectively. The surface tension of liquid Ga γ is 724 mN/m at room temperature.[2] We plot the aspect ratio by assuming $\nu = 0.5$ and $\epsilon_x^\infty = 2\epsilon_y^\infty$.

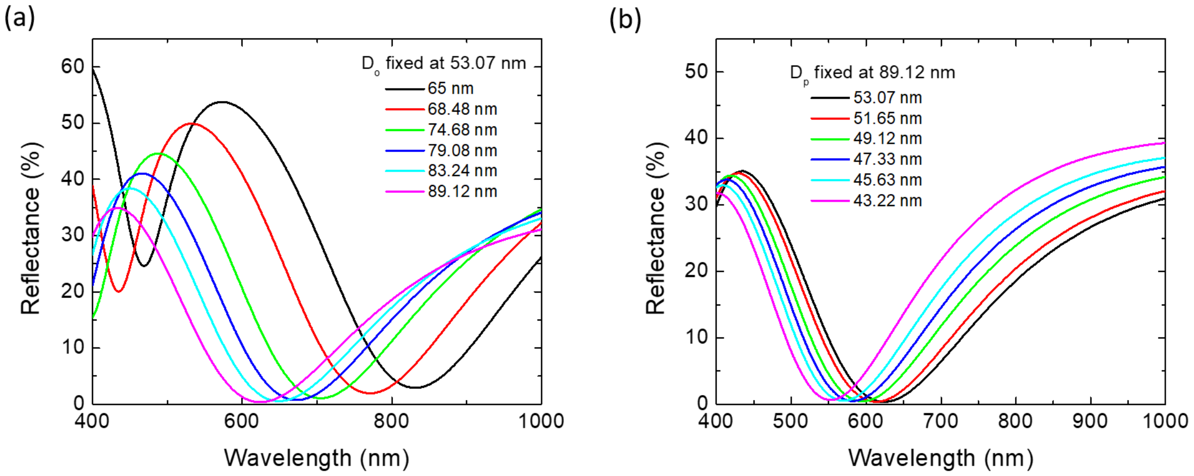

Figure S9. Simulated reflectance spectra of the three-layered NPs/PDMS structure when only considering the change in (a) D_p and (b) D_o, respectively.

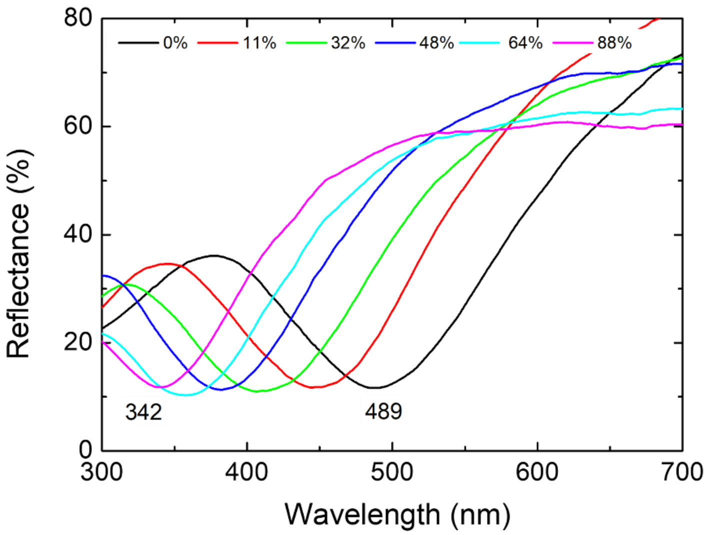

Figure S10. Measured reflectance spectra of the representative monolayered GaNPs/PDMS nanocomposite film (t_{dep} = 120 nm) under various biaxial stretch ratios.

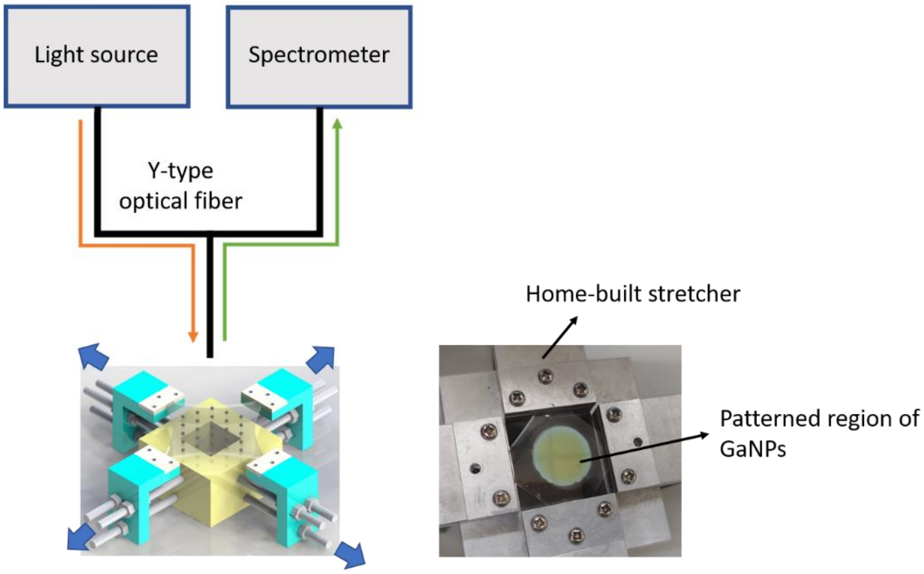

Figure S11. Schematic of the reflectance spectrum measurements for GaNPs/PDMS nanocomposites under biaxial stretching.

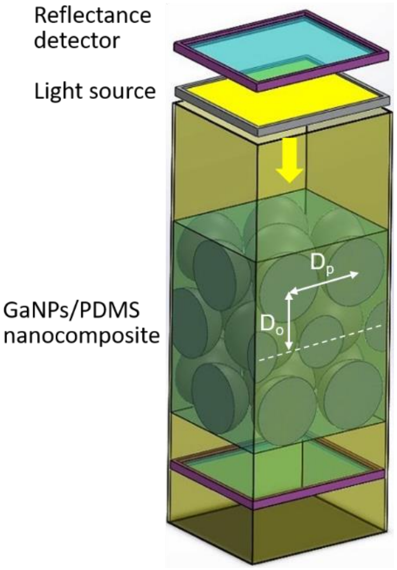

Figure S12. Hexagonal unit cell with periodic boundary conditions implemented as the computational domains in the electromagnetic modeling.

Table S1. Main parameters used in the numerical simulation.

Parameter	Description	Equation/Value
ω_p	Plasma frequency of Ga metal (eV)	14.1
D	GaNP core diameter (nm)	55
a	Interparticle distance (nm)	10.4
F_0	Maximum filling ratio of closely-packed NPs	F_0 = 0.74 for fcc
F	Filling ratio of GaNPs	$[d/(d+a)^3]F_0$
ϵ_m	Dielectric constant of surrounding medium	2.1
ϵ_∞	High-frequency dielectric constant of Ga metal	1

Table S2. Calculated intralayer spacing (D_p) and interlayer spacing (D_o) corresponding to various stretch ratios.

Stretch ratio (%)	D_p (nm)	D_o (nm)
0	65	53.07
11	68.48	51.65
32	74.68	49.12
48	79.08	47.33
64	83.24	45.63
88	89.12	43.22

References

(1) Style, R. W.; Boltyanskiy, R.; Allen, B.; Jensen, K. E.; Foote, H. P.; Wettlaufer, J. S.; Dufresne, E. R. Stiffening Solids with Liquid Inclusions. *Nat. Phys.* **2015,** *11,* 82–87.

(2) Jung, W.; Vong, M. H.; Kwon, K.; Kim, J. U.; Kwon, S. J.; Kim, T.; Dickey, M. D. Giant Decrease in Interfacial Energy of Liquid Metals by Native Oxides. *Adv. Mater.* **2024,** *36,* 2406783.
